# Editorial: Acute respiratory distress syndrome and mechanical ventilation

**DOI:** 10.3389/fmed.2022.994611

**Published:** 2022-09-20

**Authors:** Linhui Hu, Haibo Qiu, Ling Liu, Claude Guérin, Chunbo Chen

**Affiliations:** ^1^Department of Critical Care Medicine, Maoming People's Hospital, Maoming, China; ^2^Clinical Research Center, Center of Scientific Research, Maoming People's Hospital, Maoming, China; ^3^Jiangsu Provincial Key Laboratory of Critical Care Medicine, Department of Critical Care Medicine, School of Medicine, Zhongda Hospital, Southeast University, Nanjing, China; ^4^Médecine Intensive Réanimation, Hospices Civils de Lyon, Groupement Hospitalier Centre, Hôpital Edouard Herriot, Lyon, France; ^5^Department of Critical Care Medicine, Guangdong Provincial People's Hospital, Guangdong Academy of Medical Sciences, Guangzhou, China; ^6^Department of Intensive Care Unit of Cardiac Surgery, Guangdong Cardiovascular Institute, Guangdong Provincial People's Hospital, Guangdong Academy of Medical Sciences, Guangzhou, China; ^7^The Second School of Clinical Medicine, Southern Medical University, Guangzhou, China

**Keywords:** acute respiratory distress syndrome, mechanical ventilation, coronavirus disease 2019, critically ill patients, strategy, intensive care unit

Acute respiratory distress syndrome (ARDS) is a life-threatening form of respiratory failure characterized by inflammatory pulmonary edema resulting in hypoxemia (PaO_2_/FiO_2_ < 300 mmHg) (1). The heterogeneousness of ARDS substantially contributes to the complexity of its management. Mechanical ventilation (MV) is frequently used to sustain life in patients with severe ARDS, especially in the setting of coronavirus disease 2019 (COVID-19). However, a major concern in MV patients is the risk of ventilator-induced lung injury, which leads to but is partially prevented by lung-protective ventilation. However, prospective evidence, definitions, and skills all need to be developed further and shared for better implementation of personalized MV in ARDS patients with or without COVID-19. The aim of the Research Topic of the articles in this issue dedicated to critically ill patients, was to provide an overview of recent advances in ARDS and MV, and seek innovative solutions to resolve the challenges of personalized lung-protective ventilation, starting from titrating positive end-expiratory pressure (PEEP) to adjusting inspiratory trigger to weaning ventilation. Thirteen articles were submitted to this thematic collection, nine of which were original research studies, and four meta-analyses. Eleven articles are associated with COVID-19, ARDS or MV, and two articles focused on the lung physiotherapy of older sepsis patients or drug selection for anesthesia induction.

COVID-19 seriously endangers human health with ARDS and the resultant refractory hypoxemia playing as a common cause of death (2), which generally desired the use of MV with lung protection strategies. First, low tidal volume ventilation (LVTT) is recommended by major guidelines (3, 4). However, gender preference might exist in the implementation of LVTT. To compare and understand differences in the use of LTVV between females and males with ARDS related to COVID-19, Swart et al. found that in this cohort of patients, females received LTVV less often than males in the first days of invasive ventilation. The difference in the use of LTVV was mainly driven by an anthropometric factor, namely, body height. The authors suggested that use of LTVV may improve by paying attention to correct titration of tidal volume, which should be based on predicted body weight, which is a function of body height and gender.

On the other hand, appropriate PEEP setting is well-acknowledged as one of key roles to lung protection ventilation (5). However, the best way to titrate the PEEP in patients suffering from ARDS is still matter of debate. Gibot et al. conducted a pilot comparison on PEEP values derived from either electrical impedance tomography (EIT) or other techniques when ventilating patients with COVID-19. The authors found that EIT-guided PEEP personalized setting may help to achieve a more homogenous distribution of ventilation. Regarding PEEP setting in ventilated patients without ARDS, Zhou et al. conducted a Bayesian network meta-analysis and systematic review of randomized controlled trials (RCTs) comparing different levels of PEEP based on a novel classification of PEEP level to explore the optimal PEEP. The authors found that higher PEEP was associated with significantly higher PaO_2_/FiO_2_ ratio and higher incidence of pneumothorax.

When the lung function is improved, getting ventilator weaned off as soon as possible is beneficial to patient outcome (6). Spontaneous breathing trial has been used to predict the optimal time of weaning from ventilator. However, it remains controversial which trial should be preferentially selected. Yi et al. performed a meta-analysis, indicating that automatic tube compensation seems to be the optimal choice of predicting successful weaning from ventilator among critically ill patients. Jhou et al. provided evidence that proportional assist ventilation had a high probability of being the most effective ventilation mode for MV patients, regarding a higher rate of weaning success, a lower proportion of patients requiring reintubation, and a lower mortality rate than other ventilation modes. However, high quality RCTs are needed to further establish these findings.

Despite optimal ventilation and weaning strategies, ARDS is associated with high mortality. A meta-analysis by Wang et al. concluded that the incidence of ARDS in patients with burns was 24% and that mortality was as high as 31%. The incidence rates, which were related to MV, location, and inhalation injury, were significantly higher in patients from western countries than patients from Asian/African countries. To further provide reference data about risk factors for mortality in MV patients with COVID-19, Hernández-Cárdenas et al. described the clinical characteristics of mechanically ventilated COVID-19 patients in Mexico, and, by machine-learning and logistic regression models, identified that the acute kidney injury, uric acid, lactate dehydrogenase, and a longitudinal increase in the ventilatory ratio were risk factors.

Given that ARDS is associated with a high mortality and is a heterogeneous syndrome, early diagnosis that initiates early intervention, is of vital importance to expect a better prognosis. In the absence of specific early warning signals, developing biomarkers may be a way to reach this goal. As we know, ARDS is characterized by dysregulated vascular permeability. Therefore, Tanaka et al. found that plasma 5-hydroxyindoleacetic acid might be a potential biomarker of ARDS severity and highlighted the importance of evaluating vascular leakage magnitude for ARDS treatment. Meanwhile, Cheng et al. found that lower CD8 T cell count was associated with higher severity and early mortality in ARDS patients caused by *Acinetobacter baumannii* pneumonia, which could be valuable for outcome prediction.

In conclusion, the data and reviews published in this Research Topic have shown that ARDS and MV optimization strategies are very active and effective in critically ill patient, especially in the COVID-19 pandemic. However, these clinical studies are non-RCTs, and the sample sizes relatively small, making it not possible to set out any kind of recommendation, as the evidence is not yet conclusive on this Research Topic. In addition, these semantic articles do not include hot topics like extracorporeal membrane oxygenation implementation (7), non-invasive ventilation mode (8) or other hybrid approaches being considered tailored to the patients with ARDS related to COVID-19, which may open the door for the content of the next topic.

## Author contributions

LH drafted the original version of the editorial. LL, CG, HQ, and CC revised the editorial. All authors have contributed, read, and approved the final version of the manuscript.

## Conflict of interest

The authors declare that the research was conducted in the absence of any commercial or financial relationships that could be construed as a potential conflict of interest.

## Publisher's note

All claims expressed in this article are solely those of the authors and do not necessarily represent those of their affiliated organizations, or those of the publisher, the editors and the reviewers. Any product that may be evaluated in this article, or claim that may be made by its manufacturer, is not guaranteed or endorsed by the publisher.
